# The causal effects of mandatory health insurance coverage expansion in Switzerland

**DOI:** 10.1007/s10754-025-09396-5

**Published:** 2025-05-16

**Authors:** Boris Kaiser, Andreas Kohler, Christian P. R. Schmid

**Affiliations:** 1 BSS Volkswirtschaftliche Beratung, Aeschengraben 9, 4051 Basel, Switzerland; 2 ZHAW School of Manamgent and Law, Gertrudstrasse 8, 8401 Winterthur, Switzerland and Santésuisse, Lagerstrasse 107, 8004 Zürich, Switzerland; 3 University of Lucerne, Faculty of Economics and Management, and CSS Institute for Empirical Health Economics, Frohburgstrasse 3, Tribschenstrasse 21, 6002 Lucerne, Switzerland

**Keywords:** Mandatory Health Insurance, Health Care Costs, Complementary Medicine, Coverage Expansion, Physician Behaviour, Difference-in-Differences

## Abstract

The expansion of public health insurance programs affects payers as well as the behavior of service providers. In this paper, we study the expansion of Swiss mandatory health insurance in 2012 to include complementary and alternative medicine physician services. The policy change provides a quasi-experimental design that allows us to estimate the causal effects on the payer and physician behavior using a difference-in-differences framework. First, we find that from the payer’s perspective, expanding coverage to complementary and alternative medicine increases physician costs per patient by about 7 percent. Second, we find that the increase in physician service costs per patient in mandatory health insurance is almost exactly offset by a decrease in supplementary health insurance costs. Thus, suggesting that the behavior of physicians was unchanged by the coverage expansion.

## Introduction

In recent years, public health insurance programs have been expanded in many countries to either previously uncovered parts of the population or new services. For example, the expansion of Medicaid in the United States to include new parts of the population through the Patient Protection and Affordable Care Act (PPACA), or the expansion of South Korea’s National Health Insurance program to new services. Thus, it is crucial to understand, especially for policy makers, how such expansions affect payers and behavior of service providers.

In this paper, we study the expansion of mandatory health insurance (MHI) in Switzerland. In 2012, the coverage of MHI was extended to physician services in complementary and alternative medicine (CAM), in particular, anthroposophic medicine, traditional Chinese medicine, homeopathy and phytotherapy. For physicians to bill CAM services to MHI, they must have additional training in these disciplines. Prior to the reform, the costs for CAM were borne either by individuals out-of-pocket or by supplementary private health insurance (SPHI) plans. We study this reform from the perspectives of the payer and physicians by asking the following questions: (i) What are the financial consequences of coverage expansion for MHI? and (ii) How does coverage expansion affect physician behavior?

The policy change provides a quasi-experimental setting since it only affected those physicians who had acquired a diploma in CAM already *before* the policy change. The empirical strategy is based on a difference-in-differences approach in which we compare physicians with and without diplomas in CAM (i.e., treatment and control group, respectively) before and after the reform. The outcome variables of interest are measures of costs per patient at the physician level. The quasi-experimental design allows us to estimate the causal effects of the policy change.

To answer our questions, we exploit two sources of claims data that cover the period from 2007 to 2017. The first source is the so-called *Datenpool* provided by SASIS AG.^1^ It contains aggregate physician-level panel data on health care costs covered by MHI. This data covers the population of MHI costs. This first data source allows us to answer the question regarding the financial consequences for the payer. The second source is data from CSS Insurance, a health insurance company, which covers roughly one sixth of the population in MHI plans. It contains insured individuals who have *both* MHI and SPHI, with the latter covering additional ambulatory services including CAM. The latter data is only a sample. The second data source allows us to answer the question regarding physician behavior.

The main results are summarized as follows. First, we estimate that expanding the coverage to CAM increases the physician service costs per patient in MHI by approx. CHF 25 or 7%. In contrast, placebo tests show statistically insignificant effects close to zero on drug and laboratory costs per patient. Thus, being the payer, MHI bears these additional costs. Our analysis shows that these effects on MHI are largely driven by the subgroup of physicians practicing homeopathy. Second, we show that the increase in physician service costs per patient in MHI is almost exactly offset by a decrease in SPHI costs. In other words, there is no effect of coverage expansion on total physician service costs per patient. Thus, we conclude that physician behavior seems unchanged.

The broad literature on the effects of MHI expansion on health care utilization can be divided into two narrower strands of literature.

The first, and larger strand, studies the expansion of MHI coverage to parts of the population who previously had no coverage (e.g., children). This first strand often looks at individual countries such as the US in the context of the Patient Protection and Affordable Care Act including Medicaid/Medicare (e.g., Finkelstein et al., 2012; Simon et al., 2017; Mazurenko et al., 2018). These studies typically find that health care utilization increases, in particular, primary care (in terms of having a personal doctor, visits and costs) and preventive care (in terms of compliance, checkups and screening) as well as prescription drugs. Furthermore, there is evidence that physicians adjust their labor supply in terms of allocation (e.g., Garthwaite, 2012) and specialization as well as practice choices (e.g., Chen, 2018) in response to the expansion of MHI coverage. The findings on health care utilization broadly extend to other countries like lower-middle-income countries (see e.g., Erlangga et al., 2019 for a systematic review of the literature).

The second, and smaller strand, analyzes the expansion of insurance coverage to health care services that were previously not covered by MHI. There is evidence from South Korea, where all residents are enrolled in the National Health Insurance program, which has successively expanded coverage of additional services for certain patient groups (e.g., MRIs for patients with cardiovascular diseases) starting in 2013. Lee and Ko (2022) study this coverage expansion based on survey data using a difference-in-difference approach. They find that expanding coverage to previously uncovered services did not affect outpatient and hospital utilization (in terms of doctor visits). There is other evidence from the US, where in 2006, Medicare Part D was implemented for the elderly. Zhang et al., (2009) study this implementation based on claims data using segmented time-series regression models. They show that drug spending increased two years after its implementation while the evidence on monthly medical spending is mixed.

In general, we contribute to the nascent literature of the second strand by studying the causal effects of expanding coverage to additional services in MHI on the payer and service providers (i.e., physician behavior). In particular, our analysis overcomes some limitations of existing studies. First, we analyze a policy change in Switzerland that expanded coverage to CAM services in MHI to all residents and not just a subpopulation. Thus, our findings are more generalizable and more informative for the whole population compared to the existing studies of Lee and Ko (2022) and Zhang et al., (2009). Second, the policy change in Switzerland offers an ideal case study with its quasi-experimental setting. This allows us to use a difference-in-difference approach giving us more credible estimates of the causal effects compared to other methods like the segmented timeseries regression used in Zhang et al., (2009). Last, we analyze two sources of claims data covering the population and a representative subsample of this population, respectively. Therefore, our study is less likely to suffer from various forms of non-response biases and measurement error, which are often present in survey data such as used in Lee and Ko (2022).

The remainder of this paper is structured as follows. Section 2 provides institutional details on the Swiss health care system. Section 3 explains the empirical strategy and Section 4 describes the data. Section 5 presents and discusses the results, while Section 6 concludes.

## Institutional background

### The Swiss health care system

Since the comprehensive reform of the Swiss Health Insurance Law in 1996, health insurance is mandatory for all residents of Switzerland. MHI is part of the Swiss social security system and strictly regulated by the Swiss federal government. MHI plans ensure access to a comprehensive basket of health care services including outpatient physician services, inpatient care, physiotherapy, pharmaceutical drugs, laboratory tests and medical supplies. The scope of coverage is defined by the Swiss Health Insurance Law, and is the same for all plans. Health insurance companies operate under the supervision of the Federal Office of Public Health and are not allowed to make profits from selling MHI plans. In 2018, there were about 50 privately-owned insurance companies offering MHI plans, many of which also offer SPHI plans.

At the end of each year, there is an open enrollment period in which individuals can freely choose a health care plan from any recognized insurance company. Two different types of MHI plans are offered: indemnity plans, which give the insured individual a free choice of health care providers, and managed care plans, which restrict provider choice in exchange for lower premiums (e.g., Health Maintenance Organization HMO, Preferred Provider Organization PPO, or telemedicine plans). All plans contain a deductible which can be chosen from a fixed range of options (between CHF 300 and 2,500). After total out-of-pocket expenditures exceed the deductible, individuals face a co-payment rate of 10 percent up to a stop-loss amount of CHF 700.^2^ All insurance plans are individual plans, there are no family plans

Premiums are community-rated; they only vary with the type of plan, the deductible level, the place of residence (on the level of cantons or sub-canton regions) and the age group of an individual (children under 18, adolescents from 19 to 25, and adults over 26). Most state governments (i.e., cantons) provide means-tested premium subsidies to low-income residents.

On the supply side, health care providers of outpatient physician services are reimbursed fee-for-service according to a national fee schedule that is negotiated between (associations of) health service providers and insurance companies. The fee schedule is also subject to regulation by the federal government. The fee schedule ensures that *relative* prices of physician services are uniform throughout the country, however, *absolute* prices charged to patients may differ between cantons.^3^ Besides physician services, the prices for prescription drugs, diagnostic tests and medical devices are set by the federal government, and not negotiated between insurers and health service providers. In sum, since prices for products and services are fixed from the perspective of an individual physician, she can only influence her revenues through the amount and the composition of her services.

SPHI plans cover treatments and drugs beyond the coverage of MHI plans or access to private or semi-private rooms in hospitals and free choice of doctors in hospitals. In particular, SPHI plans cover additional outpatient therapies mostly offered by non-physicians. These include psychological therapy and complementary and alternative medicine (CAM) such as osteopathy, Ayurveda therapy, Bach flower remedies, art therapies, or massages. Since SPHI is voluntary, it is less strictly regulated than MHI. Thus, coverage and cost sharing varies across plans and health insurance companies. Often there is a moderate co-insurance rate between 10 and 25% coupled with an annual coverage cap typically ranging from CHF 1*,*000 to 10*,*000.

### Policy change

In 2012, MHI coverage was extended to include physician services related to the following CAM therapies: anthroposophic medicine (a holistic approach paying equal attention to body, soul and spirit), traditional Chinese medicine TCM (including medicinal herb therapy), homeopathy (a naturopathic healing method based on the so-called simila principle) and phytotherapy (a type of herbal medicine). Physicians must undergo additional training in these CAM disciplines for the treatment costs to be covered by MHI. In particular, to charge CAM treatments, a physician must obtain a diploma certifying that she has completed appropriate training. The diploma is issued by the Swiss Medical Association (FMH). The training usually includes at least 360 h of training and is concluded with an exam.^4^ Note that the services of *non-physician* therapists in these disciplines have never been covered by MHI and, as a result, were not affected by the policy change.

**Fig. 1:**
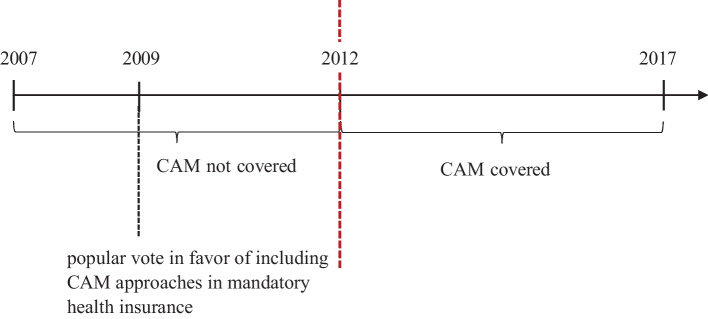
Coverage of Complementary and Alternative Medicine in Mandatory Health Insurance. *Notes:* The figure shows the time line of the policy change regarding insurance coverage of complementary and alternative medicine (CAM) in mandatory health insurance. Note that the period shown corresponds to the data availability

Before 2012, CAM practices were not covered by MHI. In May 2009, Switzerland held a popular vote on the inclusion of CAM approaches into the MHI. A majority of 67 percent of the Swiss population voted in favor. As a consequence of the popular vote in 2009, the Federal Council decided that starting from 1 January 2012 the four CAM approaches were tentatively covered by MHI for a six year trial period (see Fig. 1). While the insurance coverage was shifted from supplementary to mandatory health insurance, it is important to note that the prices for these services remained the same. Observed changes in health care costs can therefore be attributed to changes in volumes. In June 2016, the Federal Council decided to permanently include anthroposophic medicine, traditional Chinese medicine (TCM), homeopathy, and phytotherapy in MHI. Starting from 1 August 2017 those complementary health approaches are now permanently covered by MHI.^5^

## Empirical strategy

The empirical strategy consists of a difference-in-differences framework. We compare the evolution of the average health care costs before and after the policy change between two groups of primary care physicians or general practitioners (GPs): Those who had acquired a diploma in CAM already before 2012 (i.e., the treatment group) and those who never hold any diploma in CAM during the observation period (i.e., the control group).

Each physician *i* is assigned to one of the two groups. The binary variable *D*_*i*_ ∈ {0*,* 1} denotes treatment assignment, with *D*_*i*_ = 1 for the treatment group and *D*_*i*_ = 0 for the control group. Period *t* = 0 denotes the period in which the new policy becomes effective, while *t* > 0 are subsequent periods and *t* < 0 are pre-treatment periods. Each physician *i* has two *potential outcomes* for each treatment state: *Y*_*it*_(0) is the outcome of physician *i* if she belongs to the control group and *Y*_*it*_(1) is the outcome if *i* belongs to the treated group. The *observed* outcome may be written as:

1 $${Y}_{it} = {Y}_{it}(0) + {D}_{i}iI(t \ge 0)[{Y}_{it}(1) - {Y}_{it}(0)]$$

where *I*(*t* ≥ 0) is an indicator for periods affected by the treatment. We want to identify the *average treatment effect on the treated* (ATT), i.e., *τ* = *E*[*Y*_*it*_(1) − *Y*_*it*_(0)|*D*_*i*_ = 1]. This effect measures the average impact of the policy change on health care costs. Finally, we denote by *X*_*it*_ a vector of exogenous covariates.

The main identifying assumption in the difference-in-differences framework is the (conditional) parallel trends assumption, which can be stated as follows (Abadie, 2005; Lechner et al., 2011; Callaway & Sant’Anna, 2019):

2 $$E[{Y}_{it}(0) - {Y}_{i,t-1}(0)|{D}_{i} = 1, X_{i}] = E[{Y}_{it}(0) - {Y}_{i,t-1}(0)|{D}_{i} = 0, {X}_{i}],\text{ for }t \ge 0$$

The assumption states that, conditional on *X*_*it*_, trends in outcomes in the absence of the policy change run parallel across groups. Importantly, the *level* of outcomes is allowed to differ across groups, for instance, because there exist unobserved time-constant differences in GPs’ practice styles. The parallel trends assumption cannot be tested directly, but its plausibility can be assessed in a number of ways. First, if outcome trends in the pre-treatment period are largely parallel, we can be confident that the unobserved counterfactual trends in the post-treatment period would be parallel as well. Second, we can check whether some other outcome, known not to be affected by the policy change, does indeed not change in response to the treatment (i.e., a placebo test).

As Angrist and Pischke (2008) show, the difference-in-differences framework can be cast into a standard two-way fixed-effects regression:

3 $${Y}_{it} = \tau {D}_{i}I(t \ge 0) + {X}_{it}\beta + {\alpha }_{i} + {\theta }_{t} + {\varepsilon }_{it}$$

where *X*_*it*_ are time-varying covariates, *α*_*i*_ are physician-specific fixed effects, *θ*_*t*_ is a flexible nonparametric time trend and *ε*_*it*_ is an idiosyncratic error term. The interaction *D*_*i*_*I*(*t* ≥ 0) is an indicator for the treated periods in the treatment group such that *τ* captures the average treatment effect on the treated (*ATT*). The coefficients can be estimated using the within- estimator. We weigh observations by the number of patients to account for differences in practice size between physicians and across time. Standard errors are clustered at the physician-level to render inference procedures fully robust to serial correlation at the physician level (see Bertrand et al., 2004).

Time-varying covariates may be important because the policy can change the composition of patients who seek primary care. Studies show that especially young women seek CAM treatments (e.g., Melchart et al., 2005). At the same time, a change in the patient mix may affect GPs’ health care costs. Thus, we control for the following time-varying covariates at the physician- level: the share of patients in different gender and age groups (0–10, 11–20,*...*, 81–90, above 90) and the share of consultations with a high deductible (exceeding CHF 500 for adults and CHF 100 for children), which is a good indicator of patients’ health status.

## Data

To study the effects of the reform from the perspectives of the payer and physicians we use two data sets. First, we use MHI claims data from all Swiss health insurers to analyze the financial consequences of the coverage expansion for MHI as the payer. Second, to study how the reform affected the behavior of physicians we use MHI and SPHI claims data from an individual health insurer. The following subsections describe the two data sets in detail.

### Mandatory health insurance data

Aggregate annual data on mandatory health care expenditures come from the Datenpool, a nationwide database of Swiss health insurers that is operated by SASIS AG. The data cover the population of MHI claims in Switzerland (i.e., 99 percent of all MHI claims).^6^ For each physician, we observe her costs for outpatient physician services, pharmaceutical drugs, laboratory tests and medical items and devices from 2007 until 2017. We only consider primary care physicians (GPs) because CAM is mostly relevant in primary care settings. Our outcomes of interest are costs per patient at the physician level, in particular, costs for outpatient physician services. Note that a patient is defined as an insured person, who visits the physician at least once in a given year.

For each physician, we have information on her training, in particular, we know if she has a diploma in CAM practices (i.e., anthroposophical medicine, TCM, homeopathy and phytotherapy), and when she acquired it. The data on diplomas come from the federal register of medical professionals. We classify physicians into a treatment group and a control group, based on whether they hold a diploma in CAM practices. For the treatment group, we consider those physicians who acquired their diploma *before* the policy change in 2012; physicians who acquire a CAM diploma in a later year are omitted. In Fig. 5 in Appendix A we show that the number of diplomas issued between 2007 and 2017 has been relatively stable in all CAM practices.^7^ Since we have a panel data setting, we consider physicians who were active at least three years before and after 2012 to mitigate potential attrition bias. Furthermore, we drop physician-year observations with less than 50 patients, since these are mostly cases where physicians have ceased practicing medicine professionally.

### Individual health insurer data

In addition to the market data provided by SASIS, we have access to the claims data of CSS Insurance, which is the largest mandatory health insurer in Switzerland. Between 2007 and 2018, the average market share of CSS Insurance was 16%. Like all health insurers, CSS Insurance provides its MHI data to SASIS for the nationwide database. Put differently, the MHI claims data of CSS Insurance is a subset of the data described above. In contrast to the nationwide data, however, these data additionally include supplementary private health insurance (SPHI) claims. This is a crucial difference as we observe costs for CAM treatments before 2012 in these data. Hence, we can compare health care costs that consist of conventional medicine treatments *and* CAM treatments before and after the reform.

The CSS data set is constructed as follows. Using all physician identifiers from the SASIS Datenpool data, we first select all CSS Insurance customers that were treated by these physicians between 2007 and 2018. This gives us physician–patient pairs on an annual basis. Second, we keep only patients that have both a MHI plan *and* supplementary coverage for alternative medicine treatments (these are roughly 58% of all insured individuals). In other words, we ensure that costs for CAM treatments are observed before and after the policy change by excluding all potential self-payers. For the remaining physician–patient pairs, we extract all health insurance claims. Third, we aggregate health care expenditures and patient characteristics at the physician-level. We keep only physicians with at least 10 patients per year.^8^ This gives us a CSS sample of 4*,*751 physicians, which contains 270 (5*.*3%) less physicians than the SASIS Datenpool data (see Tables 1 and 4)

## Results

First, we discuss the results from the analysis of the MHI data to answer the question about the financial consequences of the reform. Second, we present the results from the analysis of the Individual Health Insurer data to answer the question how the reform affected the behavior of physicians.

### Mandatory health insurance

#### Descriptive statistics

Table 1 shows descriptive statistics for the treatment group (GPs with a certificate in CAM) and the control group (GPs without any certificate in CAM) measured in the pre-treatment period. Note that physicians with multiple CAM certificates are included in multiple subgroups. Roughly 10% of GPs have completed some training in CAM and thus belong to the treatment group. Comparing the two groups, we note considerable differences in health care costs as measured by MHI claims in the SASIS Datenpool. Physicians with CAM bill higher physician service costs per patient than their counterparts without CAM. Service costs include all diagnostic and therapeutic services offered in the physician’s office, such as physical examinations, patient counseling, keeping of medical records, x-rays, electrocardiograms, etc. Conversely, they have markedly lower costs for prescription drugs. This seems plausible as most CAM treatments are less drug-intensive than conventional medicine treatments. Comparing average patient characteristics shows that physicians in the treatment group have younger patients and a higher share of female patients, which is consistent with evidence that women display stronger preferences for CAM (see e.g., Kristoffersen et al., 2014). Regarding physician characteristics, several points are worth noting. First, the share of physicians with the specialty title ”practice medicine”, which has fewer training requirements than ”general internal medicine”, is 11 percentage points higher among the treatment group. Second, among the four sub-disciplines in CAM, TCM and homeopathy are most common, while anthroposophic medicine and phytotherapy are fairly rare. Third, physicians with training in CAM are more likely to be female and tend to run smaller practices. These two findings may be linked to the fact that female physicians more often work part-time than male physicians.

**Table 1:** Descriptive Statistics (Mandatory Health Insurance)

variable	control group		treated group	
	mean	st.dev	mean	St.dev
response variables (costs are in Swiss francs)				
physician service costs	333	157	381	198
drug costs	504	401	298	207
laboratory costs	98	56	75	60
*patient charachteristics*				
aged 0–18	10.4%	8.7%	15.9%	12.7%
aged 19–50	40.0%	11.2%	41.5%	10.0%
aged 51–65	22.0%	5.0%	22.7%	6.0%
aged 66–80	18.6%	7.3%	14.5%	6.9%
aged 81 +	9.0%	6.8%	5.3%	4.5%
female	58.4%	9.5%	64.9%	9.6%
share of high deductible	13.1%	5.2%	15.3%	5.6%
*physician characteristics*				
general internal medicine	87.8%		76.7%	
practice medicine	12.2%		23.3%	
trad. chinese medicine	-		59.2%	
homeopathy	-		33.8%	
anthroposophic medicine	-		8.9%	
phytotherapy	-		4.6%	
physician age ≤ 50	30.0%		24.0%	
physician age 51–60	40.4%		50.9%	
physician age > 60	28.7%		25.1%	
physician age unknown	0.9%		0.0%	
female physician	22.5%		36.2%	
physician sex unknown	0.1%		0.0%	
practice size	942	650	673	458
number of physicians (N)	4466		555	

This table shows descriptive statistics for the treated and control group prior to the policy change. Response variables are per patient and only include mandatory health insurance claims. For time-varying variables, the numbers are annual averages for the period 2009–2011. Standard deviations are only displayed for non-binary variables

**Fig. 2:**
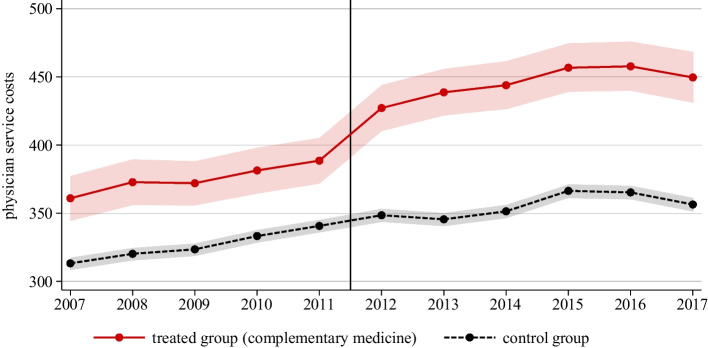
Physician Service Costs per Patient (Mandatory Health Insurance). *Notes:* The figure displays the evolution of average annual physician service costs per patient across the two treatment groups. Costs are measured in Swiss francs. The vertical line marks the policy change. Shaded areas represent 95% confidence intervals

Figure 2 shows the evolution of average annual physician service costs across the two treatment groups. Costs are measured in current Swiss francs. Prior to the policy change, the trends in average costs were remarkably steady and parallel. With the policy change in 2012 that extended coverage of MHI to include CAM practices, the costs in the treated group rose quite sharply from around 380 to 430 Swiss francs, while there was no discernible change in the control group. In other words, the increased coverage increased MHI costs at the physician level. As expected, the effect on health care costs is restricted to physician *service* costs; there are no discernible effects on either physicians’ drug prescribing costs or laboratory costs (see Figs. 6 and 7 in the Appendix).

**Fig. 3:**
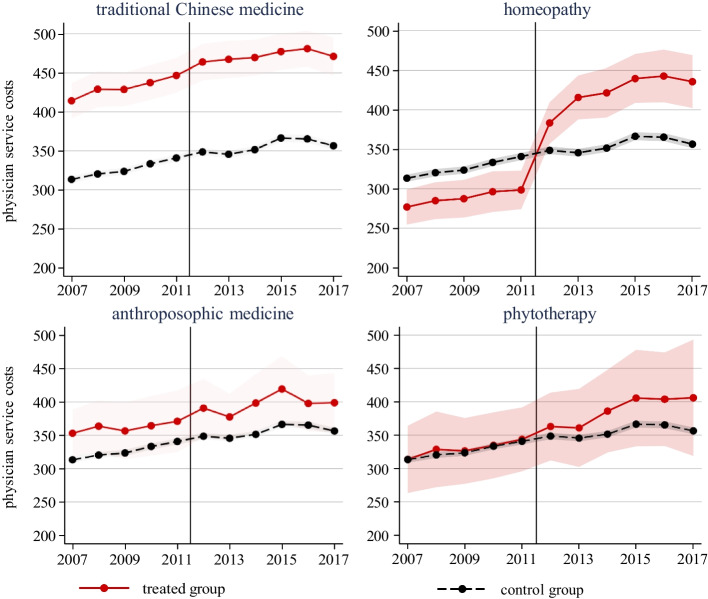
Physician Service Costs per Patient, by Specialty. *Notes:* For each specialty in complementary medicine, the figure displays the evolution of the average annual physician service costs per patient across the two treatment groups. The line in black represents the control group. The vertical line marks the policy change. Shaded areas represent 95% confidence intervals

Figure 3 shows the evolution of physician service costs for each of the four CAM disciplines separately. By far the most pronounced increase in costs per patients occurred among treated physicians with a diploma in homeopathy. For TCM or anthroposophic medicine, we see no measurable change in the data. For phytotherapy, there are too few physicians to draw meaningful inferences from the evolution of physician service costs. The results beg the question as to why these differences are so stark. First, homeopathy-practicing physicians may apply CAM more broadly to a wider range of patients, whereas the other CAM disciplines may have narrower patient target groups. Second, homeopathy-practicing physicians may counsel their patients more intensively and more repeatedly on homeopathic treatments than their counterparts in the other CAM practices.

#### Policy effect estimates

Table 2 shows the results for the outcomes physician service costs (1), drug costs (2), and laboratory costs (3) based on the estimation of Eq. (3), where the treatment group are the GPs from all four CAM disciplines. The effects on drug and laboratory costs can be considered “placebo” tests because these products and services were not affected by the coverage expansion of MHI. We see that, on average, physician service costs per patient and year increased by approximately CHF 25 or 7% in response to the policy change that is, the coverage of CAM services by MHI. By contrast, the effects on drug costs and laboratory costs are close to zero and not statistically significant.

**Table 2:** Effect of Policy Change on Costs, DiD Estimates

VARIABLES	(1) service costs	(2) drug costs	(3) lab costs
Policy effect	25.15**	2.564	−0.958
	(4.104)	(3.790)	(1.664)
Relative effect	7.1%	0.5%	−0.8%
Outcome mean	355.5	503.2	123.9
_*R*_2	0.896	0.926	0.832
Number of physicians	5053	5053	5053
Observations	53,225	53,225	53,225

DiD regressions include time and unit fixed effects and time-varying patient characteristics. Data covers time period 2007 to 2017. Cluster-robust standard errors in parentheses. Significance levels: ** p < 0.01; * p < 0.05

Table 3 shows the results for the physician service costs separately for each of the four CAM disciplines. As already suggested by Fig. 3, the policy change increased physician service costs per patient statistically significantly only for the TCM subgroup and the homeopathy subgroup. In the latter subgroup, costs per patient increased on average by CHF 70 or 20% per year.

**Table 3:** Effect of Policy Change by Treatment Subgroup, DiD Estimates

subgroups	TCM	homeopathy	anthroposophic med	phytotherapy
Policy effect	9.994*	70.81**	6.203	18.21
	(4.691)	(8.964)	(8.768)	(9.573)
Relative effect	2.8%	19.9%	1.7%	5.1%
Outcome mean	355.5	355.5	355.5	355.5
_*R*_2	0.900	0.898	0.900	0.900
Number of physicians	4826	4682	4546	4522
Observations	50,814	49,306	47,853	47,601

DiD regressions include time and unit fixed effects and time-varying patient characteristics. Data covers time period 2007 to 2017. Cluster-robust standard errors in parentheses. Significance levels: ** p < 0.01; * p < 0.05

The results in this section answer the question what the financial consequences of the reform are for MHI as the payer. Overall, GPs practicing CAM, on average, charge MHI 7% higher physician service costs per patient and year than they would have in absence of the reform.

However, these results are not informative on changes in GPs’ actual service provision to patients as CAM services were financed by other payers prior to the reform, in particular by SPHI plans. Thus, to answer the question what effect the reform had on physician behavior we next study the individual health insurer data from CSS Insurance that includes both MHI and SPHI claims.

### Effects on physician behavior

Table 4 shows descriptive statistics for the treatment and control group in the pre-treatment period. Even though the descriptive statistics in Table 4 are based on the sample of MHI and SPHI claims data from the CSS Insurance (i.e., Individual Health Insurance data) compared to the descriptive statistics in Table 1 based on the MHI data from the SASIS Datenpool (i.e., Mandatory Health Insurance data), they are remarkably similar.

**Table 4:** Descriptive Statistics (Individual Health Insurance)

	control group		treated group	
	mean	st.dev	mean	St.dev
*response variables (in Swiss francs)*				
physician service costs	334	155	380	231
drug costs	407	346	265	199
laboratory costs	84	53	75	68
alt. med. physician service costs	−	−	102	197
alt. med. drug costs	−	−	18	49
alt. med. laboratory costs	−	−	8	17
*patient characteristics*				
aged 0 − 18	26.2%		24.0%	
aged 19 − 50	33.5%		37.8%	
aged 51 − 65	18.2%		20.4%	
aged 66 − 80	15.2%		13.4%	
aged 81 +	6.9%		4.4%	
female	60.2%		66.2%	
high deductible share	16.9%		19.3%	
*physician characteristics*				
general internal medicine	89*.*1%		80*.*4%	
practice medicine	10*.*9%		19*.*6%	
trad. Chinese medicine	−		55*.*5%	
homeopathy	−		31*.*2%	
anthroposophic medicine	−		8*.*6%	
phytotherapy	−		4*.*6%	
physician age ≤ 50	33*.*9%		26*.*9%	
physician age 51 − 60	43*.*8%		50*.*6%	
physician age > 60	22*.*4%		22*.*5%	
physician age unknown	0*.*2%		0.0%	
female physician	22*.*4%		31.1%	
physician sex unknown	3*.*6%		4.8%	
practice size (no. of patients)	277*.*63	324*.*26	233*.*21	262*.*21
number of physicians (N)	4*,* 277	(95*.*8%)	474	(85*.*4%)

Based on MHI and SPHI claims data of CSS Insurance, this table shows descriptive statistics for the treated and control group prior to the policy change. Response variables are per patient; standard deviations are only displayed for non-binary variables. For time-varying variables, the numbers are annual averages for the period from 2009 to 2011. Physicians with less than 10 patients per year (i.e., less than 10 insured individuals of CSS Insurance) are excluded, which implies that the data consists of 95.8% and 85.4% of the original control and treatment group, respectively

Physician service costs, both in the control and treated group, are almost the same in the CSS Insurance data as in the SASIS Datenpool data. In contrast, drug and laboratory costs are slightly lower in the CSS Insurance data than in the SASIS Datenpool data. Patient characteristics are very similar in both data sets. However, patients in the CSS Insurance data are slightly younger in both the control and treated group (i.e., higher share of patients aged 0–18 and 19–50). Also the share of female patients and the share of patients with a high deductible are very similar. Physician characteristics in terms of physicians’ education, CAM practices, physicians’ age and sex composition are almost identical. Only in terms of practice size (i.e., number of patients) are there differences between the CSS Insurance data and the SASIS Datenpool data. Finally, sample sizes across control and treated groups are very similar (i.e., number of physicians). Overall, the descriptive statistics strongly suggest that the CSS Insurance data and the SASIS Datenpool data are comparable in the pre-treatment period.

**Fig. 4:**
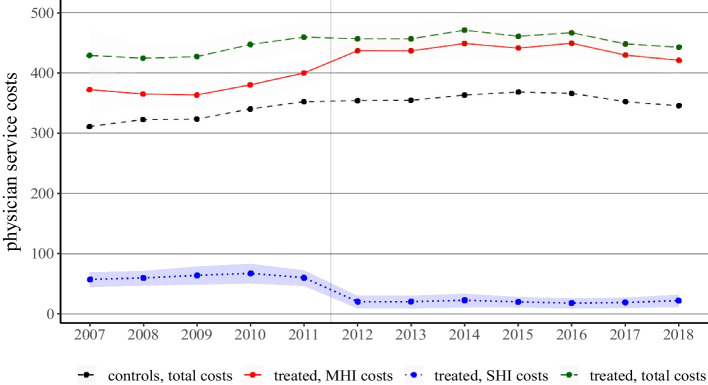
Evolution of Physician Service Costs (Individual Health Insurance). *Notes:* The figure displays the evolution of average annual physician service costs for the control group and the treatment group. For both groups, we show costs covered by mandatory health insurance (MHI); for the treatment group, we also show costs covered by supplementary private health insurance (SPHI) as well as the sum over both insurance types (total costs). Costs are measured in Swiss francs. The vertical line marks the policy change; the shaded areas represent the 95% confidence intervals

**Fig. 5:**
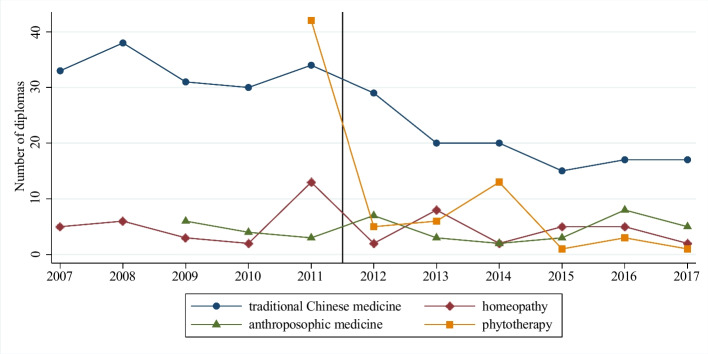
Number of CAM diplomas issued by year. *Notes:* The figure displays the evolution of new diplomas issued by the Swiss Medical Association (FMH) for each CAM discipline. It includes all primary care physicians, which were registered in the Zahlstellenregister ZSR (SASIS) and/or the Medizinalberuferegister MedReg (BAG)

**Fig. 6:**
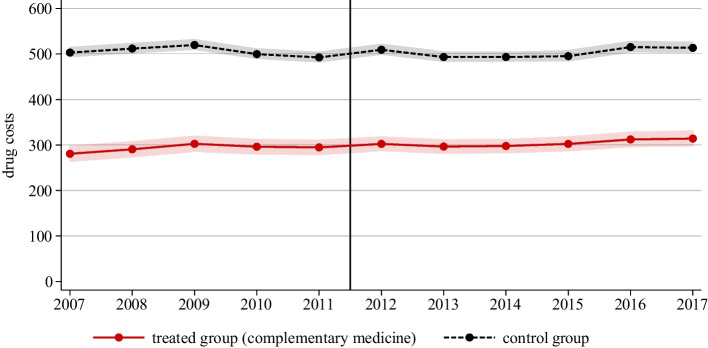
Drug Costs per Patient. *Notes:* The figure displays the evolution of average annual drug costs per patient across the two treatment groups. Costs are measured in Swiss francs. The vertical line marks the policy change. Shaded areas represent 95% confidence intervals

**Fig. 7:**
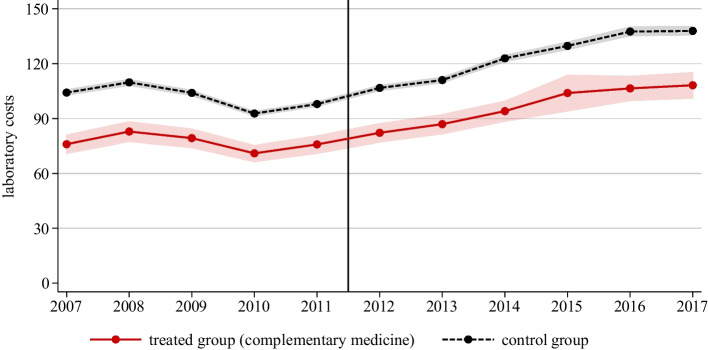
Laboratory Costs per Patient. *Notes:* The figure displays the evolution of average annual physician service costs per patient across the two treatment groups. Costs are measured in Swiss francs. The vertical line marks the policy change. Shaded areas represent 95% confidence intervals

Further similarities can be found when comparing physician service costs over time in the two data sets. Figure 4 shows the evolution of physician service costs in the control and the treatment group within the CSS data set. For the control group, we present the physician service costs covered by MHI (short-dashed line in black). In contrast, for the treatment group we present MHI costs, SPHI costs as well as total costs.

We first focus on physician service costs in the treatment group covered by MHI (solid line in red). In other words, the two lines depicting costs covered by MHI in Fig. 4 can be compared to the lines shown in Fig. 2. The evolution of physician service costs is strikingly similar. In both figures, the control group shows an upward trend until 2015 and a slight decrease after 2016; in addition, the costs in the treatment group shift noticeably upwards between 2011 and 2012 in response to the reform. In other words, the data from the two sources seem to be remarkably similar.

However, Fig. 4 shows two additional lines. First, the evolution of alternative medicine costs covered by SPHI (blue). As evident from the graph, there is a clear decline after the policy change. Note that SPHI plans already required physicians to hold a CAM certificate in order to reimburse these costs. In other words, we can add these costs to the physician service costs in the treatment group covered by MHI. Adding MHI costs and SPHI costs results in total costs (green). This line corresponds to the total physician service costs in the treatment group, that is, costs covered by both mandatory *and* supplementary health insurance. While Fig. 4 clearly indicates that total physician service costs are higher in the treatment group compared to the control group; as the evolution of (total) physician service costs in both groups seems to be parallel, it also suggests that the policy change had no effect on physician behavior. Put differently, treated physicians do not seem to increase the volume of their services. However, costs were shifted from the SPHI into the MHI.^9^

The econometric analysis corroborates the findings from the graphical analysis. Table 5 reports difference-in-differences estimates for physician service costs, drug costs, and laboratory costs. In the first column, we estimate the effect on health care costs covered by MHI. Similar to the estimate based on the full-population data in Table 2, we find an increase of roughly CHF 25 (or 7%) in physician service costs due to the policy change. In contrast, there is no statistically significant effect on drug costs an laboratory costs, which is also in line with our results above.

**Table 5:** Effect of Policy Change (CSS Insurance data, DiD estimates)

	Mandatory Ins	Supplementary Ins	Total Expenditures
Physician services	24*.*90^**^	− 32*.*67^**^	− 7*.*62
	(6*.*62)	(4*.*77)	(4*.*49)
_*R*_2	0*.*78	0*.*82	0*.*80
Outcome mean	357*.*00	2*.*50	359*.*7
Drug Costs	5*.*87	0*.*42	6*.*29
	(5*.*38)	(0*.*68)	(5*.*39)
_*R*_2	0*.*88	0*.*82	0*.*88
Outcome mean	413*.*80	0*.*60	414*.*50
Laboratory Costs	0*.*16	0*.*03	0*.*19
	(1*.*98)	(0*.*07)	(1*.*98)
_*R*_2	0*.*82	0*.*73	0*.*82
Outcome mean	104*.*90	0*.*00	105*.*00
Observations	50*,* 817	50*,* 817	50*,* 817
Number of physicians	4*,* 722	4*,* 722	4*,* 722

DiD regressions include time and unit fixed effects and time-varying patient characteristics. Data covers time period 2007 to 2018. Cluster-robust standard errors in parentheses. Significance levels: ** p < 0.01; * p < 0.05

The second column provides estimates for SPHI costs. We find a statistically negative effect in the order of minus CHF 33 for physician service costs, but a very small and statistically not significant effect on drug costs and laboratory costs. Finally, the third column shows the results for total expenditures, that is, we compare the sum of costs covered by MHI *and* SPHI of the treatment group with the costs of the control group. Here, we find no statistically significant effects; in addition, the point estimates are also small in absolute terms. Overall, our estimates suggest that the policy change did not affect the services provided by treated physicians.^10^

## Discussion and conclusion

In Switzerland, the coverage of mandatory health insurance (MHI) has been expanded to complementary and alternative medicine (CAM) in 2012. Before the reform, the costs for CAM therapies were paid by supplementary private health insurance (SPHI) or out-of-pocket by individuals. We exploit the quasi-experimental setting of this policy change using a difference-in-differences approach to analyze its financial consequences for the payer and its effect on physician behavior.

First, we find that the coverage expansion increased physician service costs per patient charged to MHI by approximately CHF 25 or 7% based on the Datenpool provided by SASIS AG, which contains the population of MHI claims. Second, we find that the coverage expansion did not change total physician service costs, that is, the sum of costs covered by MHI *and* SPHI, based on MHI and SPHI claims data provided by CSS Insurance. In other words, the policy change merely shifted costs across payers, but neither GPs nor patients seem to have adjusted their provision and consumption of CAM services in response to the policy change. On the supply side, this might be explained by the fact that (relative) prices for CAM services remained the same (i.e., fees). Furthermore, many CAM services are consumed in combination with conventional medical services limiting the possibility for physicians to induce more demand for them. On the demand side, there are two possible explanations. First, on the extensive margin, it might be that demand for CAM services is not price sensitive (see also Sect. 5.2). According to the Swiss Health Survey (Bundesamt für Statistik, 2024), the share of people who consumed CAM services has increased by 8.3 percentage points between 2002 and 2012, and by 5.7 percentage points between 2012 and 2022.

Thus, even though (relative) prices for CAM services decreased after 2012, the share of people who consumed them has not increased more than before 2012. Evidence that CAM services are consumed by a selective group of people (see e.g., Kristoffersen et al., 2014) suggests that the extensive margin of consumption is mainly driven by preferences and not by (relative) prices. Second, on the intensive margin, the out-of-pocket payments of those patients consuming CAM services may not have changed much after the policy change. According to representative surveys, about 70 percent of patients consuming CAM services have SHI plans (see Stiftung ASCA, 2013; EMR, 2024). Additionally, data from the health insurer CSS shows that the share of customers with SHI plans has not changed substantially between 2007 and 2018. This suggests that the majority of patients who consume CAM services had SHI plans before and after the policy change. Therefore, also for the majority of (actual) consumers of CAM services (relative) prices may not have changed. In other words, out-of-pocket payments were probably small before and after the policy change. Nevertheless, it is an important caveat of this paper that we cannot answer this question conclusively because we cannot include out-of-pocket payments in our empirical analysis. However, another explanation might be that consumers of CAM services are satiated with their consumption and thus do not react to decreases in (relative) prices. Our results contrast with the literature studying the expansion of MHI coverage to new parts of the population. This literature usually finds an increase in health care utilization and changes in physician behavior (see Sect. 1) whereas we find that the utilization of CAM services as well as physician behavior was unaffected by the policy change. However, our results are broadly in line with the literature studying the expansion of MHI coverage to new services. As Lee and Ko (2022), we also find no effect on health care utilization.

In multi-payer systems, where insurance coverage is split between two or more risk takers (e.g., public and private insurers), the distribution of the financial burden is of importance (e.g., Cantor, 1990; Hussey & Anderson, 2003). Our analysis suggests that the distribution of the financial burden changed in the following way. MHI premiums are lump sum premiums that are community-rated and do not (directly) depend on income. Thus, MHI premiums are highly regressive. In contrast, SPHI is optional, and individuals who are older, have more education and a higher income are more likely to choose SPHI plans (Roth et al., 2021). Furthermore, SPHI premiums are risk-rated. Hence, SPHI premiums are not or much less regressive compared to MHI premiums. This suggests that the financial burden has shifted and the financing of CAM therapies has become more regressive.

We briefly discuss potential crowding-out effects, and possible welfare consequences. A cursory glance at the CSS Insurance data on MHI and SPHI shows that the majority of people who already had SPHI plans that cover CAM services before the expansion of MHI to those services kept their supplementary health plans afterwards. This suggests that there is no crowding-out, which could incur welfare losses as individuals still pay the full SPHI premium even though the SPHI coverage was reduced. This is contrary to the literature on crowding-out effects in the case of expanding MHI to new parts of the population (e.g., Cutler & Gruber, 1996; Shore-Sheppard, 2000; Gruber & Simon, 2008; Blumberg et al., 2000; Wagner, 2015; Abraham et al., 2019; De, 2021). However, those people might only hedge themselves against the risk of CAM services being removed from MHI coverage in the future, and the risk of not being able to join a SPHI plan again. Thus, individual welfare could also remain unchanged.

There are two main lessons learned from our study from a policy perspective. First, for policy makers it is essential to know that expanding MHI coverage to new parts of the population has different effects on health care utilization than expanding MHI coverage to new services. Of course, the effects depend on the types of new services that are included. They might be different if more clinically essential services than CAM services are included. Evidence from Lee and Ko (2022) in the case of South Korea, where services like MRI scans were included, supports our findings. However, more research is needed in the future to get a more substantiated understanding. Second, expanding MHI coverage to new services shifts the financial burden from private to social financing and thus may make financing overall more regressive. At the same time, individual welfare might not change, thus creating a trade-off between financial feasibility and equity. This raises the fundamental question of which services should be covered by MHI.

## Figures and Tables

**Fig. 8:**
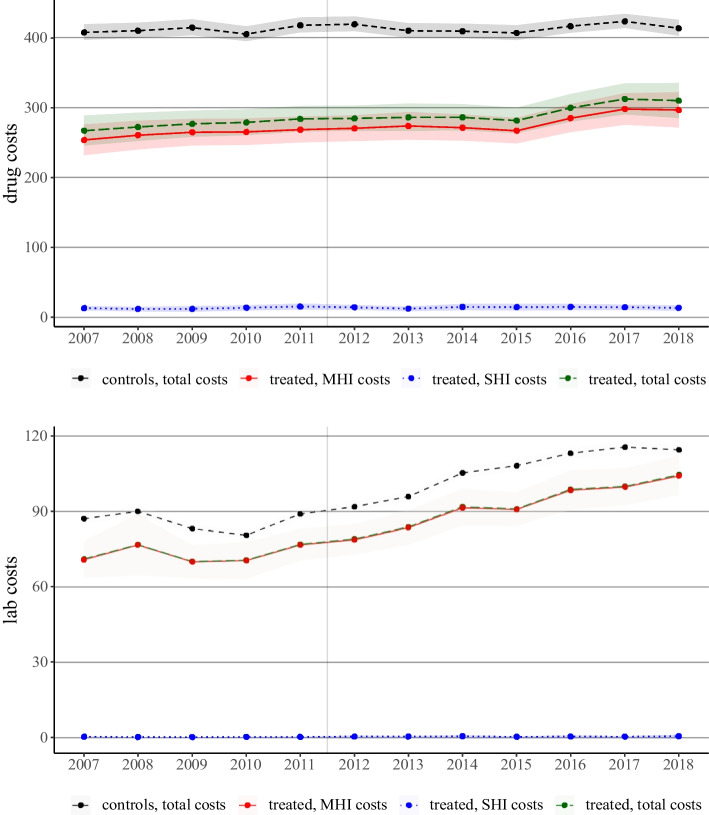
Evolution of Drug and Laboratory Costs (CSS Insurance data). *Notes:* The two figures display the evolution of average annual drug costs and laboratory costs for the control group and the treatment group. For both groups, we show costs covered by mandatory health insurance (MHI); for the treatment group, we also show costs covered by private supplementary private health insurance (SPHI) as well as the sum over both health insurance types (total costs). Costs are measured in Swiss francs. The vertical line marks the policy change; the shaded areas represent the 95% confidence intervals

**Fig. 9:**
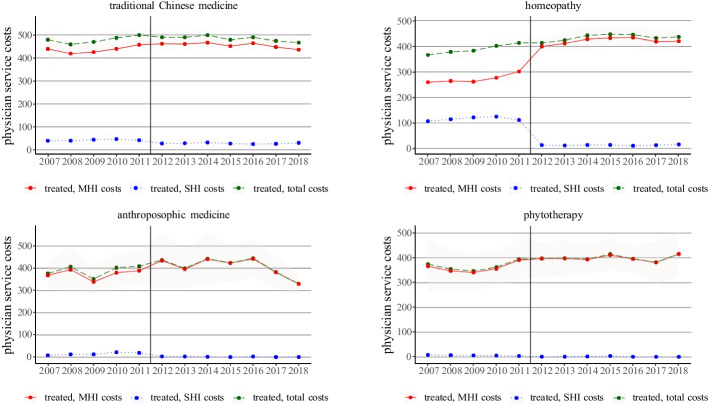
Evolution of Physician Service Costs for the Four Alternative Medicine Specializations. *Notes:* The four figures display the evolution of average annual physician service costs per patient for treatment subgroups defined by physicians’ specialization: traditional Chinese medicine (TCM), homeopathy, anthroposophic medicine, and phytotherapy. We show costs covered by mandatory health insurance (MHI); private supplementary private health insurance (SPHI) as well as the sum over both health insurance types (total costs). Costs are measured in Swiss francs. The vertical line marks the policy change; the shaded areas represent the 95% confidence intervals

## Appendix

### Additional Results

Figures 5, 6, 7, 8 and 9

## Footnotes

1 Public health authorities and insurance associations frequently rely on the Datenpool to monitor MHI costs by provider category, region and patient groups. The Datenpool is also used in research to study physician and patient behavior (see e.g., Kaiser, 2016; Burkhard, 2019; Zabrodina et al., 2020; Bischof & Kaiser, 2021).

2 For children, the deductible ranges from zero to CHF 600 and the stop-loss amount is CHF 350. If a family has more than two children insured with one insurer, the children’s cumulative stop loss amount is 700 francs.

3 To be more precise, the fees are multiplied by a conversion factor to obtain the actual prices in Swiss francs. This conversion factor is negotiated between insurers and physician organizations at the cantonal level and subject to approval by cantonal health authorities.

4
https://www.siwf.ch/weiterbildung/faehigkeitsausweise.cfm

5
https://www.admin.ch/opc/de/classified-compilation/19950219/index.html#a33

6 Note that out-of-pocket expenditures that patients do not report to their insurers are not covered by the data. However, as shown by Schmid (2017), this is a negligible issue.

7 Note that in 2011 a new program for phytotherapy was launched, and the number of diplomas issued in 2011 were most likely old diplomas previously issued under the old program (Ausweis der UNION/SMGP Phytotherapie) converted into diplomas under the new program (Faehigkeitsausweis Phytotherapie). Conversion was possible until 31 December 2021.

8 As the market share of CSS Insurance is roughly one fifth, we use here a threshold that is five times smaller than in the SASIS Datenpool data

9 As evident from Figure 9, the effects seem to be primarily driven by physicians with an additional training in homeopathy. This is in line with the results obtained using SASIS data.

10 We also took a cursory look at the demand side. There is variation in cost-sharing after the policy change due to the option for choosing different deductibles in MHI plans. Thus, we look at whether the demand response differs between individuals with high and low deductibles in their MHI plans. We find no statistically significant difference in total expenditures (i.e., SHI plus MHI expenditures) per patient between the groups of individuals with high and low deductibles. We conclude that individuals consuming CAM services might not be very sensitive to changes in (relative) prices.
